# Rapid and specific identification of emerging *Trichophyton mentagrophytes* genotype VII using an in-house developed and validated real-time PCR assay

**DOI:** 10.1128/jcm.00739-26

**Published:** 2026-09-30

**Authors:** Jinjing Zhao, Gabrielle C. Todd, Yanchun Zhu, Sudha Chaturvedi

**Affiliations:** 1 Wadsworth Center Mycology Laboratory, New York State Department of Health, Albany, New York, USA; 2 Department of Biomedical Sciences, College of Integrated Health Sciences, University at Albany 1084, Albany, New York, USA; University of Calgary, Calgary, Alberta, Canada

**Keywords:** *Trichophyton mentagrophytes *VII, real-time PCR assay

## Abstract

*Trichophyton mentagrophytes* genotype VII (*Tm*VII) is an emerging dermatophyte, primarily associated with sexual transmission, that causes persistent, painful, and inflammatory skin lesions. *Tm*VII is part of the *T. interdigitale/T. mentagrophytes* species complex, which comprises 28 genotypes. To facilitate rapid and specific identification of *Tm*VII*,* a real-time PCR (rt-PCR) assay was developed targeting three unique single-nucleotide polymorphisms in the internal transcribed spacer 1 (ITS1) region. Assay specificity was enhanced by introducing a mismatch at the 3′ ends of both primers. Using genomic DNA (gDNA) from cultured *Tm*VII isolates, the assay demonstrated high sensitivity, detecting as little as 0.0002 ng gDNA per rt-PCR. The assay was highly specific, with no cross-reactivity observed against other fungal pathogens when a cycle threshold cutoff of 37 was used. Among the 497 mold isolates tested, 47 were confirmed as *Tm*VII by rt-PCR, fully concordant with results from the standard ITS-PCR/Sanger sequencing method. The rt-PCR assay demonstrated high sensitivity, specificity, reproducibility, and speed, with a turnaround time of 1 day after DNA extraction compared with 2 to 7 days for Sanger sequencing. Future studies planned with primary skin samples in collaboration with healthcare providers are expected to further enhance the diagnostic utility of the *Tm*VII rt-PCR assay currently developed.

**IMPORTANCE:**

We developed a TaqMan probe-based real-time PCR (rt-PCR) assay to accurately identify the emerging and rapidly spreading *Trichophyton mentagrophytes* genotype VII (*Tm*VII) isolate. Timely identification and appropriate treatment of *Tm*VII infections are essential to limit their spread and improve patient outcomes. Planned studies using primary skin samples, in collaboration with healthcare providers, are expected to further enhance the diagnostic utility of the *Tm*VII rt-PCR assay.

## INTRODUCTION

*Trichophyton mentagrophytes* genotype VII (*Tm*VII) is an emerging fungal pathogen implicated in tinea corporis and pubogenitalis and is most often spread through sexual contact (1). Tinea corporis is a common skin infection caused by several types of dermatophytes, typically affecting the trunk and limbs; cases are generally mild and easy to treat (2). In contrast, tinea of pubogenital regions is rare and has been reported mostly in tropical regions (3). In 2019, a German research group identified a distinct form of tinea characterized by annularly inflamed, scaly, painful, itchy, and persistent skin lesions in 37 cases of sexually transmitted infection, which they identified as a new genotype, *T. mentagrophyte* type VII (*Tm*VII) (4). This pathogen has since spread globally to France (1), Italy (5), the United Kingdom (6), Spain (7), China (8), and the United States (9, 10).

There is an urgent need for a simple and rapid method to identify *Tm*VII to enable appropriate treatment and prevent further spread. *Tm*VII belongs to *T. interdigitale/T. mentagrophytes* species complex (*TiTm*SC), which comprises 28 genotypes, including 6 *T. interdigitale* and 22 *T. mentagrophytes* genotypes (11). Genotype-level identification is important because different *TiTm*SC members demonstrate distinct mechanisms of transmission, infection site preferences, and drug resistance profiles (11, 12). Identification to the genotype level based on conventional culture-based morphology alone is not possible because there are no unique macroscopic or microscopic features. Similarly, attempts to use matrix-assisted laser desorption/ionization-time of flight mass spectrometry (MALDI-TOF MS) to differentiate genotypes within the complex have been unsuccessful due to overlapping protein peaks (11). Alternative detection methods have been developed for dermatophyte identification including loop-mediated isothermal amplification, real-time quantitative PCR (rt-qPCR) (13), and the commercially available multiplex rt-PCR test kit DermaGenius 2.0 (14). While these methods can identify *T. mentagrophytes* to the species level, they cannot differentiate isolates to the genotype level (15). Currently, the only method for accurate *Tm*VII identification involves conventional PCR targeting the internal transcribed spacer (ITS) region, followed by Sanger sequencing and multiple alignment with ITS sequences from all 28 members of the *TiTm*SC (9). However, this process is both time-consuming and costly.

To address these limitations, we developed a highly sensitive, *Tm*VII-specific rt-PCR assay based on unique *Tm*VII single-nucleotide polymorphisms (SNPs) in the ITS1 region (11). This *Tm*VII rt-PCR assay was validated for sensitivity, specificity, and reproducibility using purified genomic (g) DNA from isolates of *Tm*VII, closely related *TiTm*SC members, and distantly related dermatophytes, molds, and yeasts. A prospective comparison of this *Tm*VII rt-PCR assay with conventional ITS-PCR/Sanger sequencing demonstrated that accurate *Tm*VII identification could be achieved within 1 day of DNA extraction, compared to 2 to 7 days for identification by the ITS-PCR/Sanger sequencing assay. This *Tm*VII rt-PCR assay represents a promising step toward developing a point-of-care assay for direct detection of *Tm*VII from skin specimens, which is expected to facilitate timely clinical decision-making and ultimately improve patient care and infection control measures.

## MATERIALS AND METHODS

### Strains and DNA

All *Tm*VII clinical isolates used in this investigation were collected from multiple healthcare facilities in New York City and surrounding counties between 2023 and 2025. Closely related isolates in the *TiTm*SC, including *T. interdigitale* I, *T. interdigitale* II, *T. interdigitale* XI, *T. mentagrophytes* II* (*Tm*II*), *T. mentagrophytes* III* (*Tm*III*), *T. mentagrophytes* IV (*Tm*IV), *T. mentagrophytes* XVIII (*Tm*XVIII), and the new species *T. indotineae* (formerly *T. mentagrophytes* VIII), as well as other dermatophyte and non-dermatophyte molds (Table S5 and S6) used in this study were identified by our in-house ITS-PCR/Sanger sequencing protocol. Yeast isolates were identified in-house by MALDI-TOF MS. DNA was extracted from dermatophytes, other molds, and yeasts, as described previously ( 12), and was stored at −80°C until use.

### Primers, probe design, and real-time PCR assay setup

A multiple alignment of the 28 genotypes of *TiTm*SC (11) revealed three unique SNPs specific to *Tm*VII within the ITS1 region (Fig. S1A). Primers were designed such that two of the SNPs unique to *Tm*VII were located at the 3′ ends of the forward (C>G) and reverse (C>T) primers. The rt-PCR probe was designed to bind the third unique SNP (C>T). To further enhance primer specificity for *Tm*VII, a mismatch was introduced at the 3′ region of the forward (“T” highlighted in green) and reverse primers (“A” highlighted in red) (Fig. S1A), as described previously (16). Primers and the probe were purchased from Integrated DNA Technologies, Inc. (Coralville, IA, USA). Sequences for primers and probe are as follows: V2670 (*Tm*VII_F1), 5′-CAGGCCGGAGGCTGGTCG-3′; V2671 (*Tm*VII_R2), 5′-GCCCGCCGAGGCAACCTAA-3′; V2672 (*Tm*VII_P), and 5′- /56 FAM/TGTGCGCCGGCCGTACCGTCC/3IABkFQ/−3′.

Primer specificity was evaluated using conventional PCR with 0.2 ng genomic DNA (gDNA) per PCR from three isolates of *Tm*VII and one isolate each of *T. interdigitale* I and II, *Tm*III*, *Tm*VI, and *T. indotineae*, with the following cycling conditions: 95°C for 60 s; 45 cycles of 95°C for 15 s, 62°C for 15 s, and 68°C for 60 s, and then a final extension at 68°C for another 5 min. The primers exclusively generated a 113-bp amplicon with gDNA from *Tm*VII (Fig. S1B); no bands were observed from any other member of the *TiTm*SC.

*Tm*VII rt-PCRs contained 1× PerfeCTa Multiplex qPCR ToughMix (Quanta Biosciences, Part # 95147-05K), 0.4 µM each of forward and reverse primers, 0.1 µM of the probe, and 2 µL of isolate DNA ranging from 0.0001 ng to 10 ng/µL in a 20 µL reaction volume. Reactions were run in an optical 96-well reaction plate (Applied Biosystems, Cat. No. 4481192) on a QuantStudio 7 Pro Dx (Applied Biosystems) with the following cycling conditions: 95°C for 60 s; 45 cycles of 95°C for 15 s, and 62°C for 50 s. Cycle threshold (Ct) values were observed only from *Tm*VII and not for any other dermatophyte tested (Fig. S1C).

### Analytical sensitivity, specificity, reproducibility, and accuracy

To evaluate the *Tm*VII rt-PCR assay sensitivity, gDNA from three isolates of *Tm*VII was serially diluted from 10 ng/µL to 0.00001 ng/µL and tested in triplicate in the rt-PCR assay on same day and over 3 different days (Table S1). Linear regression analysis was performed across six logs of gDNA concentrations. The analytical limit of detection (LOD) was determined using a probit analysis-based approach in accordance with established guidelines for molecular diagnostic assay validation. The LOD was defined as the lowest concentration at which the assay yielded ≥95% positive detection across all replicates. The slope (*s*), correlation coefficient (*R^2^*), and the amplification efficiency (*e*) of the rt-PCR assay were calculated as described previously (17).

Assay reproducibility was performed using low, medium, and high gDNA concentrations of three isolates of *Tm*VII in triplicate on the same and over 3 different days of testing (Table S2A and B). Assay specificity was determined by analyzing gDNA from 72 fungal pathogens, including members of the *TiTm*SC (*T. indotineae*, *T. interdigitale* I, *T. interdigitale* II, *T. mentagrophytes* III*, and *T. mentagrophytes* IV), other *Trichophyton* species (*T. benhamiae, T. rubrum, T. soudanense*, *T. tonsurans,* and *T. violaceum*), *Microsporum* species (*M. audouinii, M. canis*, *M. ferrugineum*, and *M. gypseum), Epidermophyton floccosum* as well as other molds and yeasts (Table S3). Assay verification was performed by testing a blinded panel consisting of 31 *Tm*VII isolates at various gDNA concentrations, with one concentration near the LOD, and 17 other molds at higher DNA concentrations (Table S4).

### Assay performance

Assay performance was evaluated using both retrospective and prospective fungal pathogens received in the laboratory. The retrospective studies included gDNA from 38 *Tm*VII isolates collected since the emergence of this genotype in New York in 2023, as well as 140 dermatophytes including *T. indotineae* (*n* = 56); *T. interdigitale* genotypes I (*n* = 2), II (*n* = 64), and XI (*n* = 1); *T. mentagrophytes* genotypes II* (*n* = 12), III (*n* = 1), IV (*n* = 1), and XVIII (*n* = 1); and other molds and yeasts (*n* = 53) (Table S5). The extracted gDNA was diluted 1:100-fold without quantification, and 2 µL of diluted gDNA was directly used in the rt-PCR. For turnaround time assessment, 266 mold isolates submitted for fungal identification by the ITS PCR/Sanger sequencing assay were prospectively tested using the *Tm*VII rt-PCR assay, following the same protocol as the retrospective study (Table S6).

### Statistical analysis

The mean, standard deviation, and percent coefficient of variance for Ct values were calculated. These calculations were performed to assess assay sensitivity, specificity, and reproducibility. The 95% confidence intervals for sensitivity and specificity were determined using the Clopper-Pearson exact method in Microsoft Excel. The LOD was statistically determined using a four-parameter logistic (4PL) regression analysis with GraphPad Prism (11.0.0, GraphPad Software, San Diego, CA, USA) and defined as the concentration yielding a ≥95% positive detection rate (18). The 4PL regression-derived LOD95 (0.000035 ng per rt-PCR) was below the lowest gDNA concentration that achieved 100% detection (0.0002 ng per rt-PCR); thus, we chose the latter concentration to be the LOD for this assay, with a cutoff of 37. A *P* value ≤ 0.05 was considered statistically significant.

## RESULTS

### *Tm*VII rt-PCR is highly sensitive, specific, and reproducible

**Fig 1:**
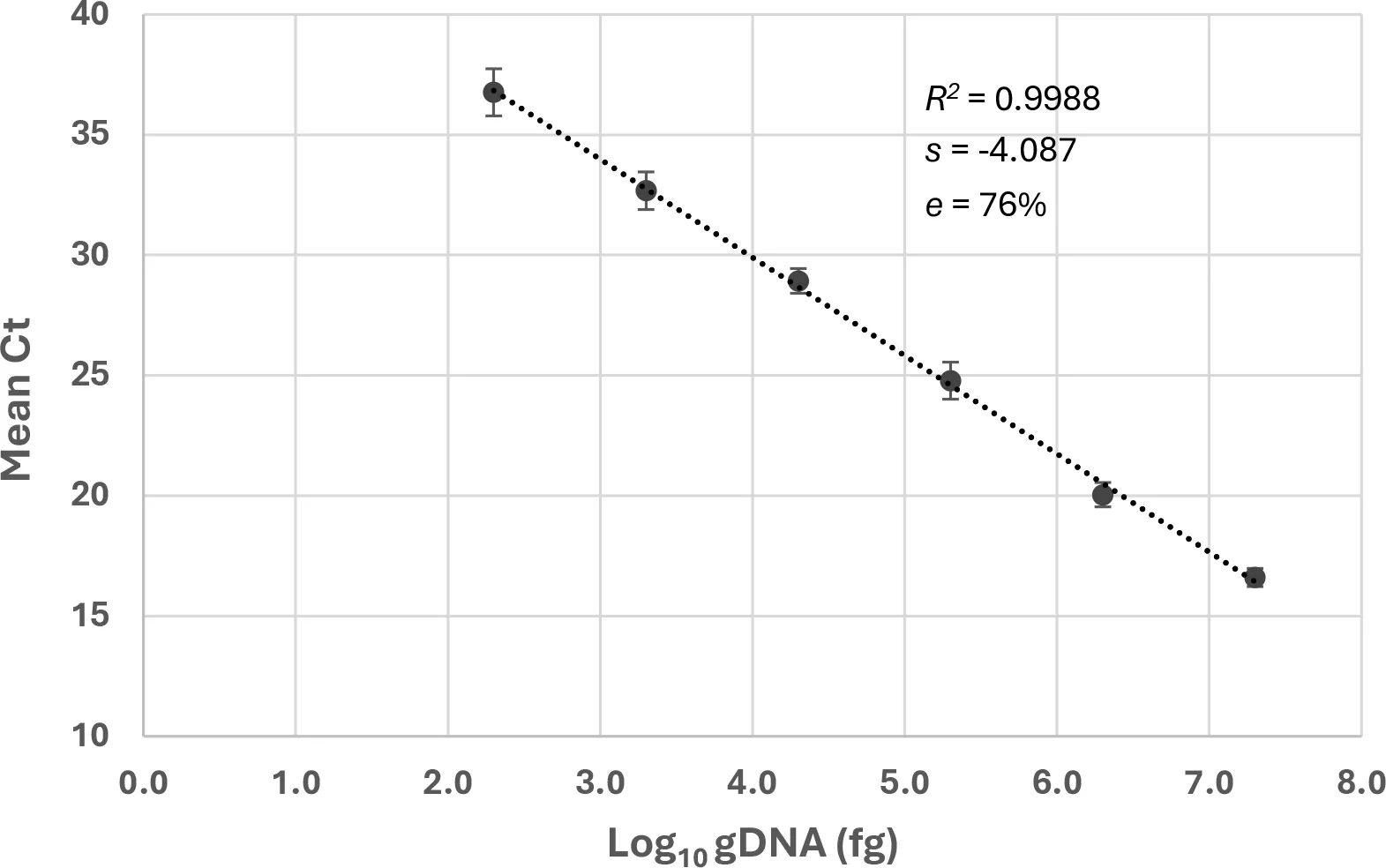
*Tm*VII rt-PCR assay sensitivity. The gDNA from three isolates of *Tm*VII was serially diluted and tested in triplicate on 3 different days. The mean *Ct* values from each dilution were plotted. The assay exhibited linearity across six orders of magnitude. The slope (*S*), correlation coefficient (*R*^2^), and amplification efficiency (*E*) are shown.

The detection rate of the *Tm*VII rt-PCR assay was calculated by testing three independent patient isolates in triplicate on 3 different days. In total, there were 27 replicates per gDNA concentration, and a Ct threshold of 37 was used to determine if *Tm*VII was present in the sample or not (Table S1). Using a 4PL regression analysis, the LOD95 was calculated as 0.000035 ng per PCR. For practical purposes, we chose the tested concentration of 0.0002 ng gDNA per rt-PCR as our LOD as 100% of samples tested at this concentration were able to detect *Tm*VII (Table S1). These results demonstrated high assay sensitivity, and the correlation coefficient (*R*^2^=0.9988) demonstrated excellent linearity across six orders of magnitude. The amplification efficiency (*e*=76%) is slightly lower than the generally accepted value of 90%; however, this does not compromise the assay’s sensitivity (Fig. 1, Table 1). The assay was highly reproducible, with consistent *Ct* values obtained for gDNA concentrations tested across three different *Tm*VII isolates within the same day and on three different days (Table S2A and S2B). The assay was highly specific, with no cross-reactivity observed to closely or distantly related fungal pathogens when a Ct cutoff of 37 was set (Table S3). Ct values exceeding 37 were interpreted as negative for *Tm*VII. In a blinded panel, the assay readily detected *Tm*VII at gDNA concentrations ranging from 0.002 to 20 ng per rt-PCR. A few dermatophytes other than *Tm*VII had low amplicon yields with Ct values above 37 at a very high gDNA concentrations (20 ng per rt-PCR). The rest of the molds and yeasts tested produced no amplicon, confirming the high accuracy of this *Tm*VII rt-PCR assay (Table S4).

**TABLE 1:** *Tm*VII rt-PCR assay performance and turnaround time^*a*^

rt-PCR (days to ID = 1)	ITS-PCR/Sanger sequencing (days to ID = 4–7)		Accuracy	Sensitivity	Specificity
	Positive	Negative			
Positive	47 (TP)	0 (FP)	100% (95% CI: 99.3%–100%)	100% (95% CI: 92.5%–100%)	100% (95% CI: 99.2%–100%)
Negative	0 (FN)	450 (TN)			

^*a*^ TP, true positive; FP, false positive; FN, false negative; TN, true negative; ID, identification.

### *Tm*VII rt-PCR revealed high performance

**Fig 2:**
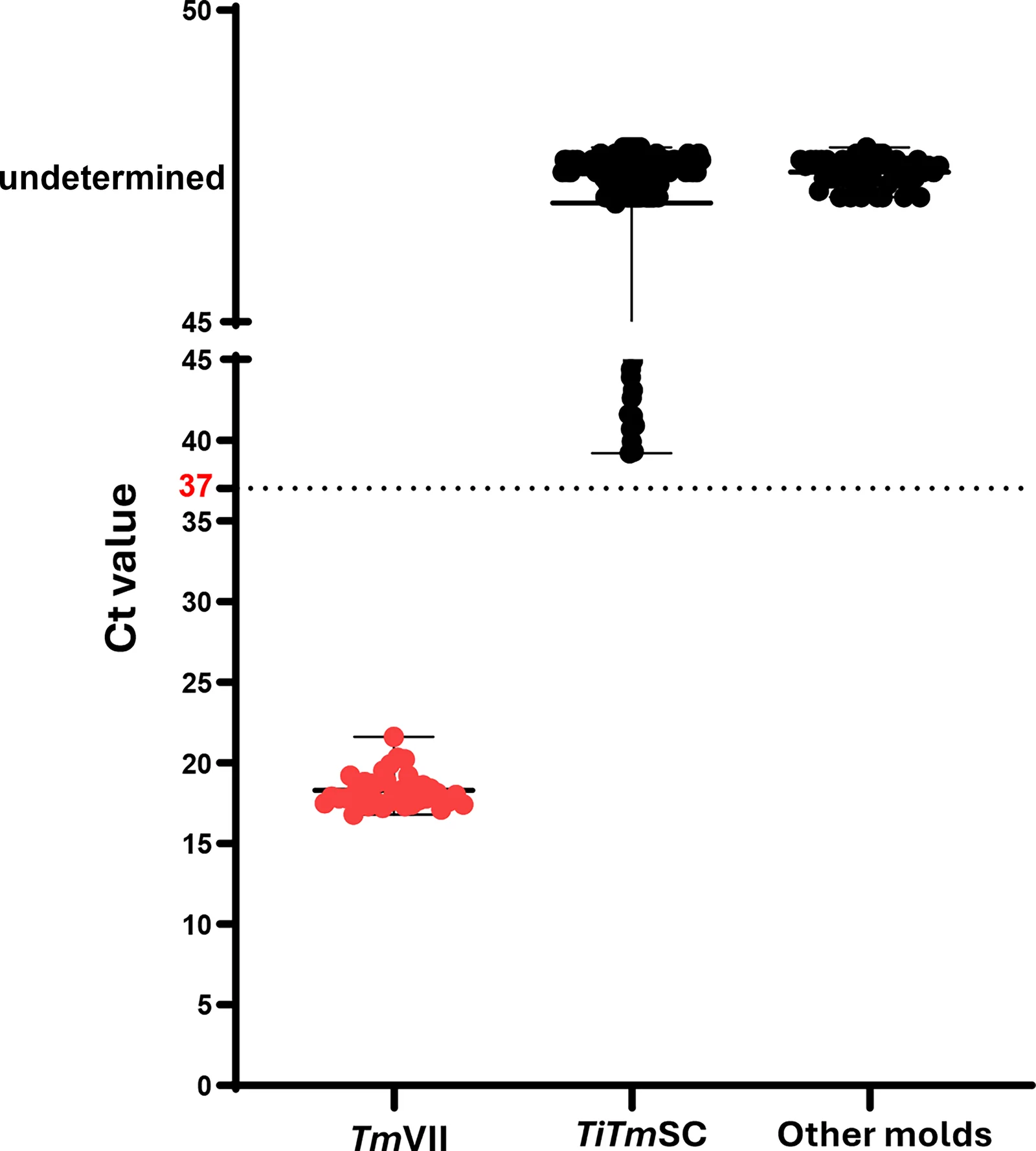
*Tm*VII rt-PCR assay performance using retrospective mold DNA samples. The gDNA from 231 molds was diluted 100-fold with water, and 2 μL of the diluted DNA was run in the rt-PCR assay in duplicate. All *Tm*VII isolates yielded Ct values below the cutoff value of 37, while a few species within the *TiTm*SC yielded Ct values above the cutoff, and other molds yielded no Ct, confirming the high degree of accuracy of the *Tm*VII rt-PCR assay.

After establishing this assay, its performance was assessed by retrospectively testing gDNA from 231 fungal pathogens and comparing rt-PCR results with the organism identification obtained by Sanger sequencing of the ITS region. Of the 231 fungal pathogens tested, 38 were positive for *Tm*VII, with Ct values ranging from 17 to 22. A limited number of other dermatophytes within the *TiTm*SC, including *T. indotineae* (*n* = 8), *T. interdigitale* I (*n* = 2), *T. interdigitale* II (*n* = 1), and *T. mentagrophytes* II* (*n* = 4), also produced Ct values ranging from 39 to 45, exceeding our established cutoff of 37 (Fig. 2 and Table S5). No other dermatophytes, other molds, or yeasts were detected by the *Tm*VII rt-PCR assay. These findings further validated our Ct cutoff value and demonstrated that the developed rt-PCR assay is highly accurate for *Tm*VII identification.

The turnaround time of the *Tm*VII rt-PCR assay was compared with that of ITS PCR/Sanger sequencing using prospective samples. A total of 266 mold isolates received between 1 July 2025 and 16 September 2025 underwent DNA extraction and were tested in the *Tm*VII rt-PCR assay. Of the 266 mold isolates, nine were identified as *Tm*VII by rt-PCR within 1 day of DNA extraction, with Ct values ranging from 16 to 29. Eight isolates produced Ct values between 40 and 45; among these, five were confirmed as *T. indotineae*, two as *T. interdigitale* II, and one each as *T. mentagrophytes* II* and *Cystobasidium slooffiae* (Table S6) by Sanger sequencing. The *Tm*VII rt-PCR assay did not cross-react with other molds. *Tm*VII identification was achieved within 1 day of gDNA extraction using rt-PCR, whereas ITS PCR/Sanger sequencing took 2 to 7 days. Overall, combining the retrospective and prospective studies, the performance of this assay was excellent, with 100% sensitivity (95% CI: 92.5%–100%) and 100% specificity (95% CI: 99.2%–100%) (Table 1). Thus, this rt-PCR assay is highly accurate, specific, and allows for the rapid identification of *Tm*VII isolates.

## DISCUSSION

*Tm*VII is an emerging dermatophyte primarily associated with sexual transmission among men who have sex with men (1, 9, 19); however, cases of household transmission involving a mother, father, and children have also been reported (20). Since its identification, *Tm*VII has spread globally (1, 5–8), raising significant concern among healthcare professionals. The first documented case of *Tm*VII infection in the United States was described in July 2024 (9), followed by several additional cases (10). Recently, *Tm*VII cases in New York have increased rapidly, suggesting possible clonal expansion of this dermatophyte (12). Due to its rapid spread, the rt-PCR assay developed in the present study will enable accurate differentiation of *Tm*VII from other genotypes within *TiTm*SC, including *T. indotineae,* facilitating timely initiation of appropriate therapy. Current literature indicates that *Tm*VII infections respond well to the first-line drug terbinafine, whereas *T. indotineae* does not (21, 22). Although they require prolonged treatments (>8 weeks), *Tm*VII is typically susceptible to terbinafine (10), especially strains circulating in New York (12). However, the first case of terbinafine-resistant *Tm*VII was reported in 2026 in a heterosexual woman from Tunisia following sexual exposure during travel to Turkey. She presented with rapidly progressive, intensely pruritic genital lesions that were unresponsive to terbinafine therapy. Antifungal susceptibility testing confirmed that the isolate recovered from this patient was resistant to terbinafine with a MIC of >8 µg/mL. The patient was subsequently treated successfully with the second-line antifungal agent itraconazole (23). This finding is concerning given the extensive global spread of terbinafine-resistant *T. indotineae* worldwide, including in the United States (12, 21).

While some rapid assays are available for the identification of *T. indotineae* (11, 16), no rapid diagnostic test is available for *Tm*VII. Additionally, *Tm*VII infections are frequently misdiagnosed as non-infectious skin conditions, such as psoriasis, or as other sexually transmitted infections (9). Delayed treatment may result in secondary bacterial infection and ongoing transmission (1). Consequently, rapid and accurate identification of *Tm*VII is vital for effective patient management and to halt the spread of this pathogen. To address this need, a TaqMan chemistry, probe-based, rt-PCR assay was developed for the rapid identification of suspected *Tm*VII isolates received in the laboratory. The assay demonstrated high sensitivity, specificity, speed, and accuracy in *Tm*VII identification.

*Tm*VII is a member of the *TiTm*SC, which comprises 28 genotypes, each with a unique ITS sequence (11). Recently, *Tm*VIII was reclassified as its own species, *T. indotineae* (24). Because whole-genome sequencing data are unavailable for all genotypes, the ITS region remains the only viable option for primer and probe design. Fortuitously, there are three unique SNPs in the ITS region of *Tm*VII that allowed for the design of primers and probes specific to this genotype. Specificity was further enhanced by introducing a mismatch at the 3′ ends of both primers, reducing the likelihood of amplification by other *TiTm*SC genotypes, a method previously utilized for *T. indotineae* identification (16). This approach, called “Polymerase Extension Block,” inhibits polymerase activity when mismatches occur in the last one to five nucleotides of the primer’s 3′ end. This mechanism serves as a barrier for polymerase extension, increasing the assay’s specificity for *Tm*VII. While this stringent design may have reduced overall PCR efficiency, it resulted in 100% specificity for *Tm*VII and did not compromise assay sensitivity. The assay produced high amplicon yields for *Tm*VII with as little as 0.0002 ng of gDNA per rt-PCR. In comparison, only low amplicon yields were observed for a few *TiTm*SC species, even when 20 ng of gDNA per rt-PCR was used; no other molds or yeast produced amplicons. Among the 497 molds tested by rt-PCR, 47 were confirmed as *Tm*VII, consistent with ITS Sanger sequencing results. A few isolates with high Ct values (over 37) were identified as other *TiTm*SC genotypes by Sanger sequencing, confirming assay specificity.

Timely recognition and appropriate treatment of *Tm*VII infections are critical for mitigating their spread and optimizing patient outcomes. The *Tm*VII rt-PCR assay for isolates yields results within 1 day of DNA extraction, compared to 2 to 7 days required for conventional ITS PCR and Sanger sequencing. This highly sensitive, specific, and rapid assay is expected to advance diagnostic capabilities, improve patient outcomes, and strengthen infection control measures. The current study is limited in scope as it utilizes isolates for identification. Therefore, future studies are planned using primary skin samples in collaboration with healthcare providers to enhance the diagnostic utility of the *Tm*VII rt-PCR assay. Until then, we recommend that clinical laboratories submit *T. mentagrophytes* isolates recovered from skin rashes, after preliminary positive laboratory tests for urease and hair perforation, to a reference laboratory with the capability of performing *T. mentagrophytes* genotyping.

## SUPPLEMENTAL MATERIAL

Fig. S1 jcm.00739-26-s0001.pdf Multiple alignment of the ITS gene, conventional, and rt-PCR.

Tables S1 to S6 jcm.00739-26-s0002.xlsx Describing assay sensitivity, specificity, reproducibility, precision, and performance evaluation.

ASM does not own the copyrights to Supplemental Material that may be linked to, or accessed through, an article. The authors have granted ASM a non-exclusive, world-wide license to publish the Supplemental Material files. Please contact the corresponding author directly for reuse.
