# Supplementary material for: Rapid and specific identification of emerging Trichophyton mentagrophytes genotype VII using an in-house developed and validated real-time PCR assay
Source: J Clin Microbiol. 2026 Sep 30;64(11):e00739-26. doi: 10.1128/jcm.00739-26 (PMC13753807; doi:10.1128/jcm.00739-26)
Supplement: Supplementary file 1 [file jcm.00739-26-s0001.pdf]

# Supplemental Fig 1

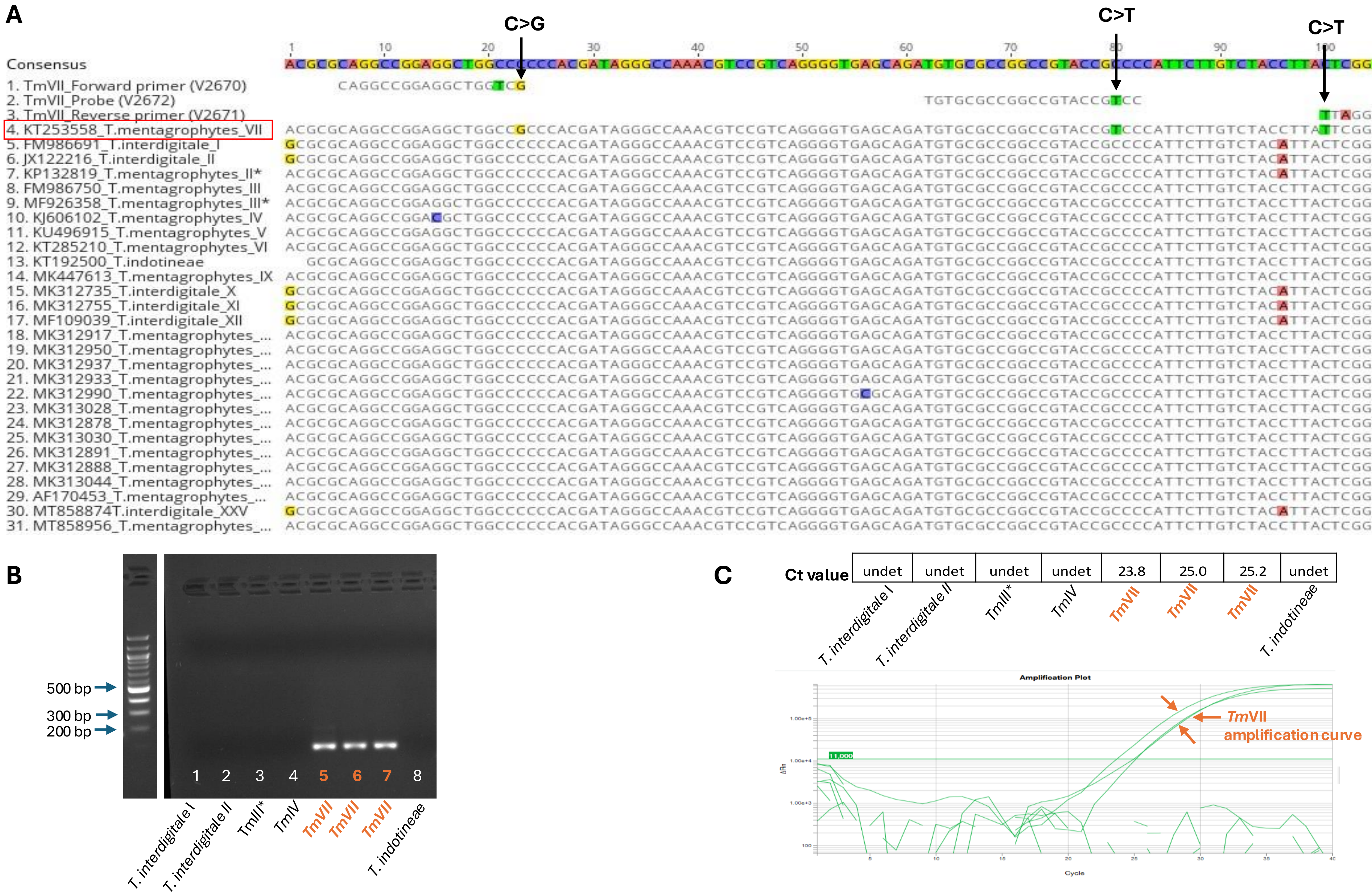

Supplemental Figure legend

**Supplemental Figure1. A)** Multiple alignment of the ITS region of all 28 genotypes in the *TiTm*SC reveal three unique SNPs (black arrows) in *Tm*VII, which were used for primer and probe design. Additionally, a mismatch was created at the 3' end of the forward (T in green) and reverse (A in red) primers to increase assay specificity. **B)** Conventional PCR with the forward and reverse primers produced a 113-bp amplicon for *Tm*VII but not for other closely related dermatophytes, confirming primer specificity. **C**) Real-time PCR yielded Ct values only for *Tm*VII, and not for other dermatophytes confirming primer and probe specificity.
